# Crystal structures of 3-meth­oxy-4-{[5-(4-meth­oxy­phen­yl)-1,3,4-oxa­diazol-2-yl]meth­oxy}benzo­nitrile and *N*-(4-{[5-(4-chloro­phen­yl)-1,3,4-oxa­diazol-2-yl]meth­oxy}phen­yl)acetamide

**DOI:** 10.1107/S2056989018016754

**Published:** 2018-11-30

**Authors:** K. Lakshmithendral, K. Archana, K. Saravanan, S. Kabilan, S. Selvanayagam

**Affiliations:** a Drug Discovery Lab, Department of Chemistry, Annamalai University, Annamalainagar, Chidambaram 608 002, India; b PG & Research Department of Physics, Government Arts College, Melur 625 106, India

**Keywords:** crystal structure, oxa­diazole derivatives, superposition, intra­molecular C—H⋯O inter­actions, inter­molecular C—H⋯N and N—H⋯N hydrogen bonds

## Abstract

The title compounds, C_18_H_15_N_3_O_4_ and C_17_H_14_ClN_3_O_3_, are heterocyclic 1,3,4-oxa­diazole derivatives which differ from each other in the groups attached to the carbon atoms: a meth­oxy­phenyl ring and a benzo­nitrile group in (I) and a chloro­phenyl ring and an acetamide group in (II). Short intra­molecular C—H⋯O hydrogen bonds occur in both mol­ecules. The crystal structure of (I) features C—H⋯N hydrogen bonds, while in the crystal structure of (II), N—H⋯N, C—H⋯N and C—H⋯O hydrogen bonds are observed.

## Abstract 2

The title heterocyclic 1,3,4-oxa­diazole derivatives differ from each other in the groups attached to the carbon atoms: a meth­oxy­phenyl ring and a benzo­nitrile group in (I) and a chloro­phenyl ring and an acetamide group in (II).

## Chemical context   

Oxa­diazole is a versatile heterocyclic nucleus, which has attracted a wide attention of the medicinal chemists for the development of new drugs. Compounds containing a heterocyclic ring system are of great importance both medicinally and industrially (Pace & Pierro, 2009 ▸). This stable and neutral hetero aromatic nucleus is associated with potent pharmacological activity that can be attributed to the presence of the toxophoric —N=C—O— linkage (Rigo & Couturier, 1985 ▸). Furthermore, 1,3,4-oxa­diazole heterocycles are very good bioisosteres of amides and esters, which can contribute substanti­ally in increasing pharmacological activity by participating in hydrogen-bonding inter­actions with the receptors (Guimaraes *et al.*, 2005 ▸). In view of the above importance of the title compounds, we have undertaken single-crystal X-ray diffraction studies for the both compounds and the results are presented here.
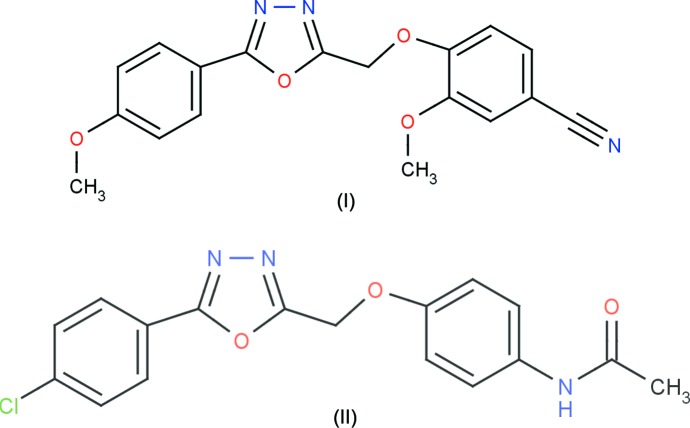


## Structural commentary   

**Figure 1:**
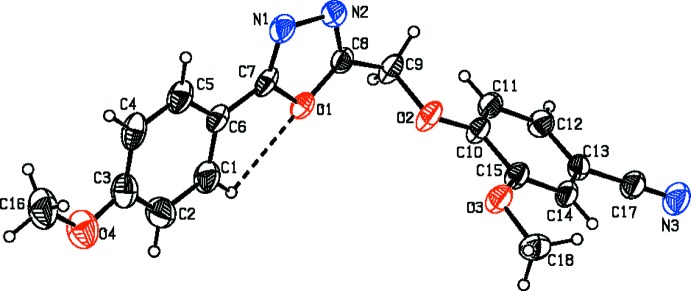
A view of the mol­ecular structure of compound (I), showing the atom labelling. Displacement ellipsoids are drawn at the 30% probability level. The dashed line represent the intra­molecular C—H⋯O inter­action (Table 1 ▸).

**Figure 3:**
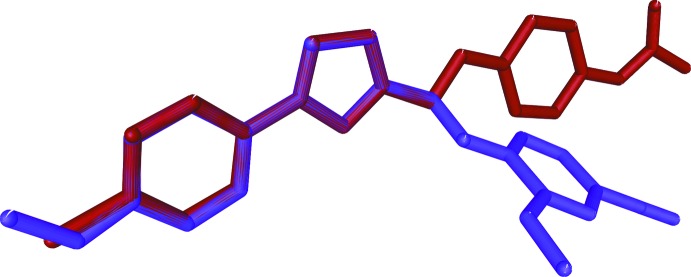
Superposition of oxa­diazole ring system of compound (I) (blue) and compound (II)(red).

The mol­ecular structures of (I) and (II) are illustrated in Figs. 1 ▸ and 2 ▸, respectively. In (I), the 4-meth­oxy­phenyl and oxa­diazole (r.m.s. deviation 0.007  Å) rings are almost coplanar with a dihedral angle of 1.4 (1)°. The meth­oxy atoms O4 and C16 are also coplanar with the rings, deviating by 0.080 (1) and 0.020 (1) Å from the mean plane of the phenyl ring, respectively. In (II), the chloro­phenyl ring is almost coplanar with the oxa­diazole ring, the angle between their mean planes being 4.0 (1)°. The whole mol­ecule is almost planar: the r.m.s. deviation is 0.098 Å and the largest deviation from the mean plane of 0.230 (2) Å is observed for atom C17. Such planarity is not observed in (I) since the meth­oxy­phenyl ring and the benzo­nitrile moiety are oriented at a dihedral angle of 66.8 (1)°. This difference can be seen in Fig. 3 ▸, which shows a superposition of the two mol­ecular structures through the oxa­diazole ring (C7/N1/N2/C8/O1) obtained using *Qmol* (Gans & Shalloway, 2001 ▸).

The mol­ecular structures of both (1) and (II) are influenced by intra­molecular C—H⋯O inter­actions (Tables 1 ▸ and 2 ▸), which form *S*(5) ring motifs (Figs. 1 ▸ and 2 ▸).

## Supra­molecular features   

**Figure 4:**
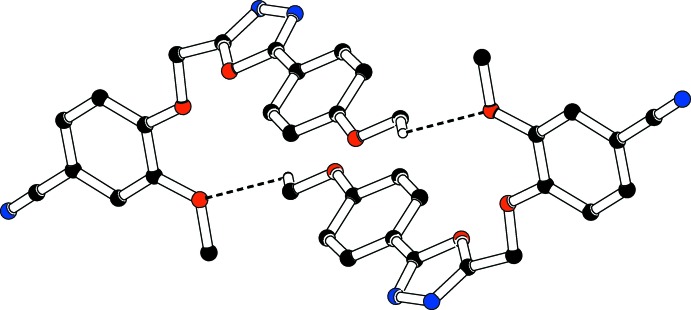
The inversion dimer formed in compound (I) via C—H⋯O inter­actions (dashed lines). For clarity H atoms not involved in these hydrogen bonds have been omitted.

**Figure 5:**
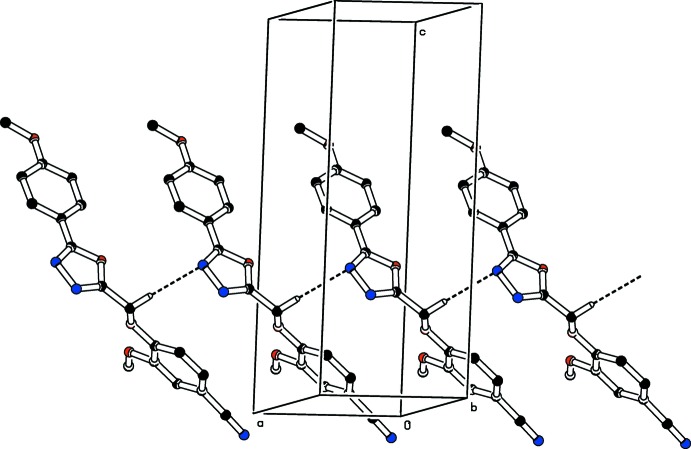
The crystal packing of compound (I) viewed down the *b* axis. The C—H⋯N hydrogen bonds (see Table 1 ▸) are shown as dashed lines. For clarity H atoms not involved in these hydrogen bonds have been omitted.

**Figure 6:**
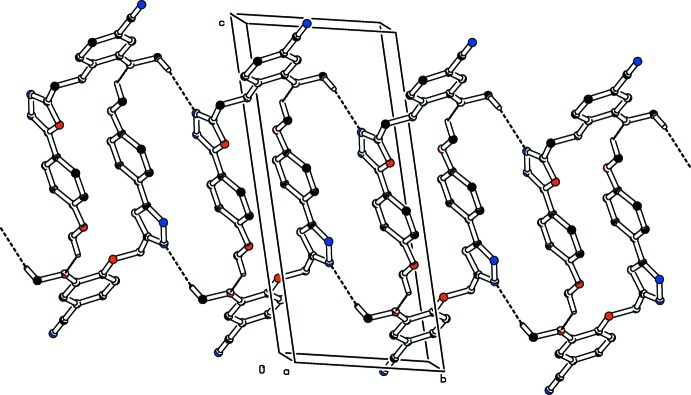
The crystal packing of the title compound (I) viewed along the *a* axis. The C—H⋯N hydrogen bonds and C—H⋯O inter­actions (see Table 1 ▸) are shown as dashed lines. For clarity H atoms not involved in these hydrogen bonds have been omitted.

In the crystal of compound (I), mol­ecules are associated via C—H⋯O inter­actions into inversion dimers (C16—H16*B*⋯O3^iii^, Table 1 ▸), generating an 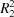
(30) motif (Fig. 4 ▸). Further C—H⋯N hydrogen bonds (C9—H9*A*⋯N1^i^, Table 1 ▸) link the mol­ecules, forming *C*(5) chains propagating along [010] (Fig. 5 ▸). There is also a weak C—H⋯N inter­action (C18—H18*A*⋯N2^ii^, Table 1 ▸) that links the mol­ecules, forming *C*(9) chains propagating in an anti-parallel manner along [110]. These C—H⋯N hydrogen bonds along with the C—H⋯O dimers form a closed cavity shape arrangement consisting of 26 atoms in the unit cell (Fig. 6 ▸). In addition, offset π–π inter­actions are observed between the centroids of inversion-related oxa­diazole and 4-meth­oxy­phenyl rings with a centroid–centroid distance of 3.700 (3) Å and a slippage of 1.037 Å.

**Figure 7:**
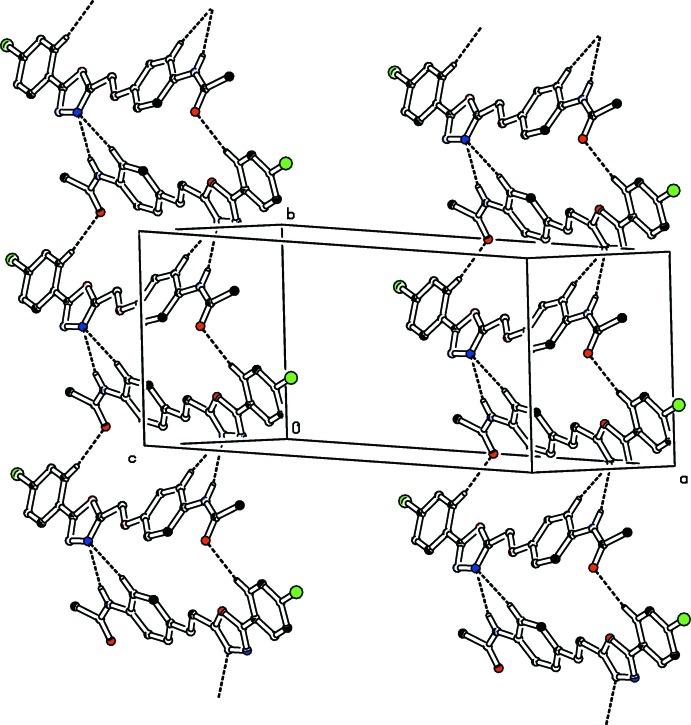
The crystal packing of (II) viewed along the *c* axis. The N—H⋯N, C—H⋯N and C—H⋯O inter­actions (see Table 2 ▸) are shown as dashed lines. For clarity H atoms not involved in these hydrogen bonds have been omitted.

In the crystal of compound (II), mol­ecules are connected by N—H⋯N hydrogen bonds forming *C*(10) chains, C—H⋯N hydrogen bonds forming *C*(8) chains and C—H⋯O inter­actions forming *C*(15) chains (Fig. 7 ▸). All these chains propagate along [010] in a helical manner. In addition, C—H⋯O inter­actions involving atoms H17*C* and O3 are also observed (Table 2 ▸). No π–π inter­actions are observed in compound (II) because of the coplanarity between the oxa­diazole and chloro­phenyl rings.

## Synthesis and crystallization   

Compound (I) was synthesized from a solution of 4-hy­droxy-3-meth­oxy­benzo­nitrile (1mmol), K_2_CO_3_ (3 mmol) in DMF (4 mL), 2-(chloro­meth­yl)-5-(4-meth­oxy­phen­yl)-1,3,4-oxa­diazole and KI (0.5 mmol). The reaction mixture was stirred at room temperature for about 2 h until the starting material had been consumed (TLC monitoring), and then washed with cold water. The solid product was collected by filtration and dried under vacuum. The pure compound was further recrystallized from ethyl acetate/petroleum ether solution (*v*:*v* = 1:1).

Compound (II) was synthesized from a solution of *N*-(4-hy­droxy­phen­yl)acetamide (1mmol), K_2_CO_3_ (3 mmol) in ACN (5mL), 2-(chloro­meth­yl)-5-(4-chloro­phen­yl)-1,3,4-oxa­diazole and KI (0.5 mmol). The reaction mixture was stirred under reflux condition for about 16 h, until completion of the reaction (TLC monitoring), then it was diluted with ethyl acetate (30 mL) and washed with saturated NaHCO_3_ and cold water. The organic layer was separated, dried over anhydrous Na_2_SO_4_ and concentrated under vacuum. The pure compound was further recrystallized from an ethyl acetate/petroleum ether solution (*v*:*v* = 1:1), giving colourless block-like crystals suitable for X-ray diffraction analysis.

## Refinement   

Crystal data, data collection and structure refinement details are summarized in Table 3 ▸. In both crystal structures, H atoms were placed in idealized positions and allowed to ride on their parent atoms: C—H = 0.93–0.97 Å with *U*_iso_(H) = 1.5*U*_eq_(C-methyl) and 1.2*U*_eq_(C) for other H atoms.

## Supplementary Material

Crystal structure: contains datablock(s) I, II, global. DOI: 10.1107/S2056989018016754/zq2243sup1.cif

Structure factors: contains datablock(s) I. DOI: 10.1107/S2056989018016754/zq2243Isup2.hkl

Structure factors: contains datablock(s) II. DOI: 10.1107/S2056989018016754/zq2243IIsup4.hkl

Supporting information file. DOI: 10.1107/S2056989018016754/zq2243Isup4.cml

Supporting information file. DOI: 10.1107/S2056989018016754/zq2243IIsup5.cml

CCDC references: 1881075, 1881074

Additional supporting information:  crystallographic information; 3D view; checkCIF report

## Figures and Tables

**Figure 2:**
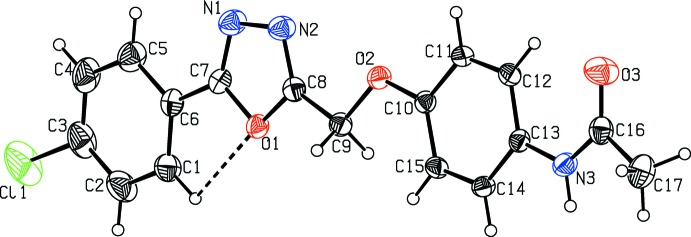
A view of the mol­ecular structure of compound (II), showing the atom labelling. Displacement ellipsoids are drawn at the 30% probability level. The dashed line represents the intra­molecular C—H⋯O inter­action (Table 2 ▸).

**Table 1:** Hydrogen-bond geometry (Å, °) for (I)

*D*—H⋯*A*	*D*—H	H⋯*A*	*D*⋯*A*	*D*—H⋯*A*
C1—H1⋯O1	0.93	2.50	2.835 (6)	101
C9—H9*A*⋯N1^i^	0.97	2.57	3.540 (6)	178
C18—H18*A*⋯N2^ii^	0.96	2.60	3.470 (6)	151
C16—H16*B*⋯O3^iii^	0.96	2.59	3.094 (7)	113

Symmetry codes: (i) 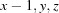; (ii) 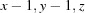; (iii) 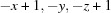.

**Table 2:** Hydrogen-bond geometry (Å, °) for (II)

*D*—H⋯*A*	*D*—H	H⋯*A*	*D*⋯*A*	*D*—H⋯*A*
C1—H1⋯O1	0.93	2.54	2.853 (3)	100
N3—H3⋯N2^i^	0.86	2.55	3.377 (3)	161
C1—H1⋯O3^i^	0.93	2.46	3.359 (3)	163
C14—H14⋯N2^i^	0.93	2.59	3.443 (3)	152
C17—H17*C*⋯O3^ii^	0.96	2.56	3.357 (4)	140

Symmetry codes: (i) 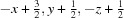; (ii) 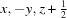.

**Table 3:** Experimental details

	(I)	(II)
Crystal data		
Chemical formula	C_18_H_15_N_3_O_4_	C_17_H_14_ClN_3_O_3_
*M*_r_	337.33	343.76
Crystal system, space group	Triclinic, *P* 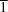	Monoclinic, *C*2/*c*
Temperature (K)	298	298
*a*, *b*, *c* (Å)	6.0847 (14), 8.5048 (19), 17.286 (4)	42.24 (1), 10.233 (3), 7.496 (2)
α, β, γ (°)	102.668 (7), 90.646 (6), 109.813 (8)	90, 91.016 (11), 90
*V* (Å^3^)	817.5 (3)	3239.6 (15)
*Z*	2	8
Radiation type	Mo *K*α	Mo *K*α
μ (mm^−1^)	0.10	0.26
Crystal size (mm)	0.24 × 0.21 × 0.19	0.22 × 0.20 × 0.18

Data collection		
Diffractometer	Bruker SMART APEX CCD area-detector	Bruker SMART APEX CCD area-detector
No. of measured, independent and observed [*I* > 2σ(*I*)] reflections	4701, 3593, 1137	8758, 3650, 2733
*R*_int_	0.087	0.117
(sin θ/λ)_max_ (Å^−1^)	0.650	0.649

Refinement		
*R*[*F* ^2^ > 2σ(*F* ^2^)], *wR*(*F* ^2^), *S*	0.086, 0.282, 0.95	0.063, 0.182, 1.05
No. of reflections	3593	3650
No. of parameters	228	218
H-atom treatment	H-atom parameters constrained	H-atom parameters constrained
Δρ_max_, Δρ_min_ (e Å^−3^)	0.49, −0.33	0.25, −0.36

Computer programs: *SMART* and *SAINT* (Bruker, 2002 ▸), *SHELXS97* (Sheldrick, 2008 ▸), *SHELXL2018* (Sheldrick, 2015 ▸), *ORTEP-3 for Windows* (Farrugia, 2012 ▸) and *PLATON* (Spek, 2009 ▸).

## supplementary crystallographic information

### 3-Methoxy-4-{[5-(4-methoxyphenyl)-1,3,4-oxadiazol-2-yl]methoxy}benzonitrile (I) Crystal data

**Table:**

C_18_H_15_N_3_O_4_	*Z* = 2
*M**_r_* = 337.33	*F*(000) = 352
Triclinic, *P*1	*D*_x_ = 1.370 Mg m^−^^3^
*a* = 6.0847 (14) Å	Mo *K*α radiation, λ = 0.71073 Å
*b* = 8.5048 (19) Å	Cell parameters from 3118 reflections
*c* = 17.286 (4) Å	θ = 3.2–27.4°
α = 102.668 (7)°	µ = 0.10 mm^−^^1^
β = 90.646 (6)°	*T* = 298 K
γ = 109.813 (8)°	Block, colourless
*V* = 817.5 (3) Å^3^	0.24 × 0.21 × 0.19 mm

### 3-Methoxy-4-{[5-(4-methoxyphenyl)-1,3,4-oxadiazol-2-yl]methoxy}benzonitrile (I) Data collection

**Table:**

Bruker SMART APEX CCD area-detector diffractometer	*R*_int_ = 0.087
Radiation source: fine-focus sealed tube	θ_max_ = 27.5°, θ_min_ = 3.1°
ω and φ scans	*h* = −7→7
4701 measured reflections	*k* = −9→10
3593 independent reflections	*l* = −22→14
1137 reflections with *I* > 2σ(*I*)	

### 3-Methoxy-4-{[5-(4-methoxyphenyl)-1,3,4-oxadiazol-2-yl]methoxy}benzonitrile (I) Refinement

**Table:**

Refinement on *F*^2^	0 restraints
Least-squares matrix: full	Hydrogen site location: inferred from neighbouring sites
*R*[*F*^2^ > 2σ(*F*^2^)] = 0.086	H-atom parameters constrained
*wR*(*F*^2^) = 0.282	*w* = 1/[σ^2^(*F*_o_^2^) + (0.0956*P*)^2^] where *P* = (*F*_o_^2^ + 2*F*_c_^2^)/3
*S* = 0.95	(Δ/σ)_max_ < 0.001
3593 reflections	Δρ_max_ = 0.49 e Å^−^^3^
228 parameters	Δρ_min_ = −0.32 e Å^−^^3^

### 3-Methoxy-4-{[5-(4-methoxyphenyl)-1,3,4-oxadiazol-2-yl]methoxy}benzonitrile (I) Special details

**Table:**

Geometry. All esds (except the esd in the dihedral angle between two l.s. planes) are estimated using the full covariance matrix. The cell esds are taken into account individually in the estimation of esds in distances, angles and torsion angles; correlations between esds in cell parameters are only used when they are defined by crystal symmetry. An approximate (isotropic) treatment of cell esds is used for estimating esds involving l.s. planes.

### 3-Methoxy-4-{[5-(4-methoxyphenyl)-1,3,4-oxadiazol-2-yl]methoxy}benzonitrile (I) Fractional atomic coordinates and isotropic or equivalent isotropic displacement parameters (Å^2^)

**Table:**

	*x*	*y*	*z*	*U*_iso_*/*U*_eq_	
O1	0.1843 (5)	0.2454 (3)	0.36727 (18)	0.0573 (8)	
O2	−0.1120 (5)	0.0785 (3)	0.21803 (19)	0.0672 (10)	
O3	−0.2533 (6)	−0.2488 (4)	0.16208 (19)	0.0661 (10)	
O4	0.6234 (7)	0.1389 (5)	0.6784 (2)	0.0999 (13)	
N1	0.5537 (7)	0.3888 (5)	0.3540 (2)	0.0666 (11)	
N2	0.4067 (7)	0.3864 (5)	0.2876 (2)	0.0679 (12)	
N3	−1.0776 (8)	−0.3032 (5)	−0.0393 (3)	0.0818 (14)	
C1	0.3016 (9)	0.1713 (6)	0.5111 (3)	0.0746 (15)	
H1	0.145930	0.131974	0.490115	0.089*	
C2	0.3548 (10)	0.1334 (7)	0.5810 (3)	0.0810 (17)	
H2	0.235535	0.069914	0.606852	0.097*	
C3	0.5834 (10)	0.1892 (7)	0.6124 (3)	0.0737 (15)	
C4	0.7621 (9)	0.2890 (7)	0.5738 (3)	0.0744 (15)	
H4	0.917781	0.329479	0.594902	0.089*	
C5	0.7024 (9)	0.3258 (6)	0.5045 (3)	0.0734 (14)	
H5	0.820509	0.392725	0.479518	0.088*	
C6	0.4759 (7)	0.2674 (5)	0.4710 (3)	0.0534 (11)	
C7	0.4163 (7)	0.3047 (5)	0.3975 (3)	0.0563 (12)	
C8	0.1976 (8)	0.3015 (5)	0.2998 (3)	0.0510 (11)	
C9	−0.0194 (8)	0.2584 (5)	0.2476 (3)	0.0617 (13)	
H9A	−0.132615	0.297492	0.277473	0.074*	
H9B	0.015621	0.314695	0.203787	0.074*	
C10	−0.3104 (7)	0.0114 (5)	0.1662 (3)	0.0524 (11)	
C11	−0.4371 (8)	0.1049 (5)	0.1460 (3)	0.0559 (12)	
H11	−0.387569	0.222949	0.167178	0.067*	
C12	−0.6385 (8)	0.0239 (6)	0.0940 (3)	0.0572 (12)	
H12	−0.723785	0.087491	0.080049	0.069*	
C13	−0.7123 (8)	−0.1506 (6)	0.0632 (2)	0.0531 (11)	
C14	−0.5881 (7)	−0.2481 (5)	0.0840 (2)	0.0545 (12)	
H14	−0.640032	−0.366455	0.063238	0.065*	
C15	−0.3888 (8)	−0.1680 (5)	0.1354 (3)	0.0545 (12)	
C16	0.8476 (11)	0.1995 (8)	0.7151 (4)	0.110 (2)	
H16A	0.939257	0.139919	0.684979	0.165*	
H16B	0.842598	0.180561	0.767860	0.165*	
H16C	0.917417	0.320492	0.718006	0.165*	
C17	−0.9170 (9)	−0.2349 (6)	0.0064 (3)	0.0621 (13)	
C18	−0.3336 (9)	−0.4316 (5)	0.1354 (3)	0.0741 (15)	
H18A	−0.454760	−0.482003	0.166887	0.111*	
H18B	−0.205201	−0.471141	0.140725	0.111*	
H18C	−0.394768	−0.464801	0.080479	0.111*	

### 3-Methoxy-4-{[5-(4-methoxyphenyl)-1,3,4-oxadiazol-2-yl]methoxy}benzonitrile (I) Atomic displacement parameters (Å^2^)

**Table:**

	*U*^11^	*U*^22^	*U*^33^	*U*^12^	*U*^13^	*U*^23^
O1	0.0402 (18)	0.0536 (18)	0.059 (2)	−0.0017 (14)	−0.0116 (15)	0.0045 (15)
O2	0.062 (2)	0.0410 (16)	0.081 (2)	0.0082 (15)	−0.0299 (18)	−0.0021 (16)
O3	0.065 (2)	0.0469 (18)	0.075 (2)	0.0105 (15)	−0.0204 (18)	0.0094 (16)
O4	0.087 (3)	0.126 (3)	0.086 (3)	0.039 (3)	−0.016 (2)	0.023 (3)
N1	0.044 (2)	0.072 (3)	0.061 (3)	−0.002 (2)	0.002 (2)	0.003 (2)
N2	0.059 (3)	0.069 (3)	0.055 (3)	0.000 (2)	0.004 (2)	0.010 (2)
N3	0.078 (3)	0.074 (3)	0.078 (3)	0.013 (3)	−0.027 (3)	0.010 (2)
C1	0.063 (3)	0.078 (3)	0.068 (3)	0.014 (3)	−0.021 (3)	0.009 (3)
C2	0.066 (4)	0.098 (4)	0.073 (4)	0.015 (3)	−0.003 (3)	0.029 (3)
C3	0.074 (4)	0.083 (4)	0.065 (3)	0.037 (3)	−0.007 (3)	0.005 (3)
C4	0.049 (3)	0.089 (4)	0.070 (4)	0.025 (3)	−0.021 (3)	−0.010 (3)
C5	0.047 (3)	0.078 (3)	0.073 (4)	0.009 (3)	−0.011 (3)	−0.004 (3)
C6	0.042 (3)	0.049 (2)	0.054 (3)	0.007 (2)	−0.005 (2)	−0.005 (2)
C7	0.035 (2)	0.047 (2)	0.064 (3)	0.002 (2)	−0.006 (2)	−0.013 (2)
C8	0.054 (3)	0.041 (2)	0.048 (3)	0.012 (2)	0.002 (2)	−0.002 (2)
C9	0.063 (3)	0.041 (2)	0.069 (3)	0.013 (2)	−0.020 (3)	−0.004 (2)
C10	0.048 (3)	0.039 (2)	0.059 (3)	0.009 (2)	−0.013 (2)	0.002 (2)
C11	0.062 (3)	0.043 (2)	0.057 (3)	0.014 (2)	−0.008 (2)	0.008 (2)
C12	0.051 (3)	0.058 (3)	0.062 (3)	0.018 (2)	−0.005 (2)	0.014 (2)
C13	0.049 (3)	0.058 (3)	0.041 (2)	0.007 (2)	−0.002 (2)	0.007 (2)
C14	0.055 (3)	0.048 (2)	0.050 (3)	0.009 (2)	−0.011 (2)	0.004 (2)
C15	0.051 (3)	0.045 (2)	0.060 (3)	0.013 (2)	−0.013 (2)	0.006 (2)
C16	0.093 (5)	0.145 (6)	0.077 (4)	0.033 (4)	−0.019 (4)	0.015 (4)
C17	0.063 (3)	0.053 (3)	0.061 (3)	0.013 (2)	−0.011 (3)	0.008 (2)
C18	0.087 (4)	0.049 (3)	0.087 (4)	0.020 (3)	0.006 (3)	0.022 (3)

### 3-Methoxy-4-{[5-(4-methoxyphenyl)-1,3,4-oxadiazol-2-yl]methoxy}benzonitrile (I) Geometric parameters (Å, º)

**Table:**

O1—C8	1.347 (5)	C5—H5	0.9300
O1—C7	1.381 (5)	C6—C7	1.446 (6)
O2—C10	1.368 (5)	C8—C9	1.480 (6)
O2—C9	1.409 (5)	C9—H9A	0.9700
O3—C15	1.371 (5)	C9—H9B	0.9700
O3—C18	1.426 (5)	C10—C11	1.371 (5)
O4—C3	1.350 (6)	C10—C15	1.409 (5)
O4—C16	1.375 (6)	C11—C12	1.383 (6)
N1—C7	1.274 (5)	C11—H11	0.9300
N1—N2	1.439 (5)	C12—C13	1.373 (6)
N2—C8	1.278 (5)	C12—H12	0.9300
N3—C17	1.146 (5)	C13—C14	1.392 (5)
C1—C2	1.380 (6)	C13—C17	1.445 (6)
C1—C6	1.396 (6)	C14—C15	1.369 (5)
C1—H1	0.9300	C14—H14	0.9300
C2—C3	1.370 (7)	C16—H16A	0.9600
C2—H2	0.9300	C16—H16B	0.9600
C3—C4	1.409 (7)	C16—H16C	0.9600
C4—C5	1.377 (6)	C18—H18A	0.9600
C4—H4	0.9300	C18—H18B	0.9600
C5—C6	1.369 (6)	C18—H18C	0.9600
			
C8—O1—C7	102.6 (3)	C8—C9—H9B	110.0
C10—O2—C9	117.8 (3)	H9A—C9—H9B	108.4
C15—O3—C18	116.4 (3)	C11—C10—O2	124.7 (4)
C3—O4—C16	119.1 (5)	C11—C10—C15	119.8 (4)
C7—N1—N2	106.0 (4)	O2—C10—C15	115.4 (3)
C8—N2—N1	105.4 (4)	C10—C11—C12	120.2 (4)
C2—C1—C6	121.6 (5)	C10—C11—H11	119.9
C2—C1—H1	119.2	C12—C11—H11	119.9
C6—C1—H1	119.2	C13—C12—C11	119.8 (4)
C1—C2—C3	120.0 (5)	C13—C12—H12	120.1
C1—C2—H2	120.0	C11—C12—H12	120.1
C3—C2—H2	120.0	C12—C13—C14	120.9 (4)
O4—C3—C2	116.6 (5)	C12—C13—C17	120.1 (4)
O4—C3—C4	123.8 (5)	C14—C13—C17	119.0 (4)
C2—C3—C4	119.5 (5)	C15—C14—C13	119.3 (4)
C5—C4—C3	118.9 (5)	C15—C14—H14	120.4
C5—C4—H4	120.5	C13—C14—H14	120.4
C3—C4—H4	120.5	O3—C15—C14	125.3 (4)
C6—C5—C4	122.5 (5)	O3—C15—C10	114.7 (4)
C6—C5—H5	118.8	C14—C15—C10	120.0 (4)
C4—C5—H5	118.8	O4—C16—H16A	109.5
C5—C6—C1	117.5 (5)	O4—C16—H16B	109.5
C5—C6—C7	121.9 (5)	H16A—C16—H16B	109.5
C1—C6—C7	120.7 (4)	O4—C16—H16C	109.5
N1—C7—O1	112.4 (4)	H16A—C16—H16C	109.5
N1—C7—C6	128.2 (4)	H16B—C16—H16C	109.5
O1—C7—C6	119.4 (4)	N3—C17—C13	179.0 (6)
N2—C8—O1	113.6 (4)	O3—C18—H18A	109.5
N2—C8—C9	127.0 (4)	O3—C18—H18B	109.5
O1—C8—C9	119.4 (4)	H18A—C18—H18B	109.5
O2—C9—C8	108.3 (3)	O3—C18—H18C	109.5
O2—C9—H9A	110.0	H18A—C18—H18C	109.5
C8—C9—H9A	110.0	H18B—C18—H18C	109.5
O2—C9—H9B	110.0		
			
C7—N1—N2—C8	0.3 (5)	C7—O1—C8—N2	−0.5 (5)
C6—C1—C2—C3	−0.5 (8)	C7—O1—C8—C9	178.1 (3)
C16—O4—C3—C2	−176.1 (5)	C10—O2—C9—C8	−178.1 (4)
C16—O4—C3—C4	5.9 (8)	N2—C8—C9—O2	114.2 (5)
C1—C2—C3—O4	−176.3 (5)	O1—C8—C9—O2	−64.2 (5)
C1—C2—C3—C4	1.7 (8)	C9—O2—C10—C11	−5.7 (7)
O4—C3—C4—C5	176.8 (5)	C9—O2—C10—C15	176.9 (4)
C2—C3—C4—C5	−1.1 (8)	O2—C10—C11—C12	−178.7 (4)
C3—C4—C5—C6	−0.7 (8)	C15—C10—C11—C12	−1.4 (7)
C4—C5—C6—C1	1.8 (7)	C10—C11—C12—C13	0.4 (7)
C4—C5—C6—C7	−179.0 (4)	C11—C12—C13—C14	0.6 (7)
C2—C1—C6—C5	−1.2 (7)	C11—C12—C13—C17	−177.4 (4)
C2—C1—C6—C7	179.6 (4)	C12—C13—C14—C15	−0.6 (7)
N2—N1—C7—O1	−0.7 (5)	C17—C13—C14—C15	177.5 (4)
N2—N1—C7—C6	179.2 (4)	C18—O3—C15—C14	−2.0 (7)
C8—O1—C7—N1	0.8 (5)	C18—O3—C15—C10	176.9 (4)
C8—O1—C7—C6	−179.2 (4)	C13—C14—C15—O3	178.5 (4)
C5—C6—C7—N1	1.4 (7)	C13—C14—C15—C10	−0.4 (7)
C1—C6—C7—N1	−179.5 (4)	C11—C10—C15—O3	−177.6 (4)
C5—C6—C7—O1	−178.7 (4)	O2—C10—C15—O3	−0.1 (6)
C1—C6—C7—O1	0.5 (6)	C11—C10—C15—C14	1.4 (7)
N1—N2—C8—O1	0.1 (5)	O2—C10—C15—C14	178.9 (4)
N1—N2—C8—C9	−178.3 (4)		

### 3-Methoxy-4-{[5-(4-methoxyphenyl)-1,3,4-oxadiazol-2-yl]methoxy}benzonitrile (I) Hydrogen-bond geometry (Å, º)

**Table:**

*D*—H···*A*	*D*—H	H···*A*	*D*···*A*	*D*—H···*A*
C1—H1···O1	0.93	2.50	2.835 (6)	101
C9—H9*A*···N1^i^	0.97	2.57	3.540 (6)	178
C18—H18*A*···N2^ii^	0.96	2.60	3.470 (6)	151
C16—H16*B*···O3^iii^	0.96	2.59	3.094 (7)	113

Symmetry codes: (i) *x*−1, *y*, *z*; (ii) *x*−1, *y*−1, *z*; (iii) −*x*+1, −*y*, −*z*+1.

### N-(4-{[5-(4-Chlorophenyl)-1,3,4-oxadiazol-2-yl]methoxy}phenyl)acetamide (II) Crystal data

**Table:**

C_17_H_14_ClN_3_O_3_	*F*(000) = 1424
*M**_r_* = 343.76	*D*_x_ = 1.410 Mg m^−^^3^
Monoclinic, *C*2/*c*	Mo *K*α radiation, λ = 0.71073 Å
*a* = 42.24 (1) Å	Cell parameters from 5528 reflections
*b* = 10.233 (3) Å	θ = 3.3–27.2°
*c* = 7.496 (2) Å	µ = 0.26 mm^−^^1^
β = 91.016 (11)°	*T* = 298 K
*V* = 3239.6 (15) Å^3^	Block, colourless
*Z* = 8	0.22 × 0.20 × 0.18 mm

### N-(4-{[5-(4-Chlorophenyl)-1,3,4-oxadiazol-2-yl]methoxy}phenyl)acetamide (II) Data collection

**Table:**

Bruker SMART APEX CCD area-detector diffractometer	*R*_int_ = 0.117
Radiation source: fine-focus sealed tube	θ_max_ = 27.5°, θ_min_ = 3.1°
ω and φ scans	*h* = −52→54
8758 measured reflections	*k* = −13→12
3650 independent reflections	*l* = −6→9
2733 reflections with *I* > 2σ(*I*)	

### N-(4-{[5-(4-Chlorophenyl)-1,3,4-oxadiazol-2-yl]methoxy}phenyl)acetamide (II) Refinement

**Table:**

Refinement on *F*^2^	0 restraints
Least-squares matrix: full	Hydrogen site location: inferred from neighbouring sites
*R*[*F*^2^ > 2σ(*F*^2^)] = 0.063	H-atom parameters constrained
*wR*(*F*^2^) = 0.182	*w* = 1/[σ^2^(*F*_o_^2^) + (0.0635*P*)^2^ + 1.9656*P*] where *P* = (*F*_o_^2^ + 2*F*_c_^2^)/3
*S* = 1.05	(Δ/σ)_max_ = 0.002
3650 reflections	Δρ_max_ = 0.25 e Å^−^^3^
218 parameters	Δρ_min_ = −0.36 e Å^−^^3^

### N-(4-{[5-(4-Chlorophenyl)-1,3,4-oxadiazol-2-yl]methoxy}phenyl)acetamide (II) Special details

**Table:**

Geometry. All esds (except the esd in the dihedral angle between two l.s. planes) are estimated using the full covariance matrix. The cell esds are taken into account individually in the estimation of esds in distances, angles and torsion angles; correlations between esds in cell parameters are only used when they are defined by crystal symmetry. An approximate (isotropic) treatment of cell esds is used for estimating esds involving l.s. planes.

### N-(4-{[5-(4-Chlorophenyl)-1,3,4-oxadiazol-2-yl]methoxy}phenyl)acetamide (II) Fractional atomic coordinates and isotropic or equivalent isotropic displacement parameters (Å^2^)

**Table:**

	*x*	*y*	*z*	*U*_iso_*/*U*_eq_	
Cl1	1.01226 (2)	0.28663 (15)	−0.00562 (17)	0.1223 (5)	
O1	0.85574 (3)	0.18095 (14)	0.0915 (2)	0.0407 (4)	
O2	0.77454 (3)	0.07687 (14)	0.0967 (2)	0.0425 (4)	
O3	0.62394 (4)	0.0180 (2)	0.2485 (3)	0.0729 (6)	
N1	0.86735 (5)	−0.00435 (19)	−0.0435 (3)	0.0519 (5)	
N2	0.83462 (4)	−0.00355 (19)	−0.0042 (3)	0.0487 (5)	
N3	0.65271 (4)	0.19545 (18)	0.3324 (3)	0.0446 (4)	
H3	0.651157	0.270429	0.383345	0.053*	
C1	0.91898 (6)	0.2770 (3)	0.0713 (4)	0.0552 (6)	
H1	0.903405	0.332106	0.114707	0.066*	
C2	0.95026 (7)	0.3187 (3)	0.0652 (4)	0.0723 (8)	
H2	0.955862	0.401652	0.105345	0.087*	
C3	0.97283 (6)	0.2357 (4)	−0.0008 (4)	0.0742 (9)	
C4	0.96543 (6)	0.1127 (4)	−0.0627 (4)	0.0737 (8)	
H4	0.981049	0.058641	−0.108061	0.088*	
C5	0.93441 (6)	0.0710 (3)	−0.0564 (4)	0.0623 (7)	
H5	0.929027	−0.011804	−0.097927	0.075*	
C6	0.91116 (5)	0.1521 (2)	0.0118 (3)	0.0462 (5)	
C7	0.87852 (5)	0.1050 (2)	0.0164 (3)	0.0417 (5)	
C8	0.82940 (5)	0.1064 (2)	0.0735 (3)	0.0392 (5)	
C9	0.79968 (5)	0.1607 (2)	0.1473 (3)	0.0395 (5)	
H9A	0.801380	0.166062	0.276309	0.047*	
H9B	0.795908	0.247872	0.100672	0.047*	
C10	0.74482 (5)	0.11247 (18)	0.1555 (3)	0.0345 (4)	
C11	0.72024 (5)	0.02966 (19)	0.1083 (3)	0.0376 (4)	
H11	0.724354	−0.043669	0.039235	0.045*	
C12	0.68942 (5)	0.05388 (19)	0.1623 (3)	0.0393 (5)	
H12	0.673114	−0.002716	0.129254	0.047*	
C13	0.68317 (5)	0.16400 (19)	0.2665 (3)	0.0356 (4)	
C14	0.70774 (5)	0.24918 (19)	0.3082 (3)	0.0377 (4)	
H14	0.703636	0.324139	0.374048	0.045*	
C15	0.73839 (5)	0.22421 (19)	0.2532 (3)	0.0376 (4)	
H15	0.754604	0.282438	0.281810	0.045*	
C16	0.62573 (5)	0.1230 (2)	0.3258 (3)	0.0481 (5)	
C17	0.59772 (6)	0.1809 (3)	0.4218 (4)	0.0633 (7)	
H17A	0.579119	0.174637	0.346840	0.095*	
H17B	0.601896	0.271026	0.448890	0.095*	
H17C	0.594398	0.133676	0.530577	0.095*	

### N-(4-{[5-(4-Chlorophenyl)-1,3,4-oxadiazol-2-yl]methoxy}phenyl)acetamide (II) Atomic displacement parameters (Å^2^)

**Table:**

	*U*^11^	*U*^22^	*U*^33^	*U*^12^	*U*^13^	*U*^23^
Cl1	0.0409 (4)	0.1918 (13)	0.1346 (10)	−0.0285 (6)	0.0098 (5)	−0.0347 (9)
O1	0.0337 (7)	0.0388 (7)	0.0495 (9)	0.0005 (6)	0.0009 (6)	0.0002 (6)
O2	0.0347 (7)	0.0388 (7)	0.0541 (9)	−0.0004 (6)	0.0022 (6)	−0.0049 (6)
O3	0.0530 (10)	0.0679 (12)	0.0983 (15)	−0.0194 (10)	0.0142 (10)	−0.0212 (11)
N1	0.0459 (10)	0.0484 (11)	0.0618 (12)	0.0004 (9)	0.0093 (9)	−0.0053 (9)
N2	0.0445 (10)	0.0434 (10)	0.0583 (12)	−0.0042 (8)	0.0070 (8)	−0.0058 (8)
N3	0.0391 (9)	0.0393 (9)	0.0554 (11)	0.0024 (8)	0.0032 (8)	−0.0038 (8)
C1	0.0397 (12)	0.0612 (15)	0.0648 (15)	−0.0029 (11)	0.0036 (11)	−0.0073 (12)
C2	0.0495 (15)	0.085 (2)	0.082 (2)	−0.0147 (14)	−0.0027 (14)	−0.0151 (17)
C3	0.0378 (13)	0.112 (3)	0.0733 (19)	−0.0085 (15)	0.0046 (12)	−0.0051 (18)
C4	0.0419 (13)	0.097 (2)	0.083 (2)	0.0130 (15)	0.0095 (13)	−0.0038 (17)
C5	0.0491 (13)	0.0675 (16)	0.0708 (17)	0.0051 (12)	0.0107 (12)	−0.0023 (13)
C6	0.0377 (11)	0.0514 (12)	0.0495 (12)	0.0033 (10)	0.0006 (9)	0.0048 (10)
C7	0.0389 (11)	0.0408 (11)	0.0453 (11)	0.0062 (9)	0.0024 (8)	0.0030 (9)
C8	0.0394 (10)	0.0375 (10)	0.0407 (10)	−0.0029 (8)	−0.0008 (8)	0.0062 (8)
C9	0.0372 (10)	0.0380 (10)	0.0433 (11)	−0.0016 (8)	0.0000 (8)	0.0017 (8)
C10	0.0357 (10)	0.0328 (9)	0.0349 (9)	0.0008 (8)	−0.0011 (7)	0.0032 (7)
C11	0.0414 (10)	0.0306 (9)	0.0407 (10)	0.0013 (8)	−0.0034 (8)	−0.0029 (8)
C12	0.0356 (10)	0.0363 (10)	0.0458 (11)	−0.0044 (8)	−0.0033 (8)	−0.0015 (8)
C13	0.0344 (10)	0.0338 (9)	0.0385 (10)	0.0017 (8)	−0.0009 (8)	0.0034 (8)
C14	0.0430 (11)	0.0306 (9)	0.0393 (10)	0.0014 (8)	−0.0002 (8)	−0.0023 (8)
C15	0.0376 (10)	0.0329 (9)	0.0423 (11)	−0.0061 (8)	−0.0027 (8)	−0.0011 (8)
C16	0.0398 (11)	0.0518 (13)	0.0529 (13)	−0.0011 (10)	0.0007 (9)	0.0055 (10)
C17	0.0422 (13)	0.0750 (18)	0.0729 (17)	0.0050 (12)	0.0125 (12)	0.0086 (14)

### N-(4-{[5-(4-Chlorophenyl)-1,3,4-oxadiazol-2-yl]methoxy}phenyl)acetamide (II) Geometric parameters (Å, º)

**Table:**

Cl1—C3	1.746 (3)	C5—C6	1.390 (3)
O1—C8	1.354 (2)	C5—H5	0.9300
O1—C7	1.366 (2)	C6—C7	1.461 (3)
O2—C10	1.386 (2)	C8—C9	1.488 (3)
O2—C9	1.412 (2)	C9—H9A	0.9700
O3—C16	1.222 (3)	C9—H9B	0.9700
N1—C7	1.292 (3)	C10—C11	1.381 (3)
N1—N2	1.419 (3)	C10—C15	1.387 (3)
N2—C8	1.288 (3)	C11—C12	1.392 (3)
N3—C16	1.359 (3)	C11—H11	0.9300
N3—C13	1.423 (3)	C12—C13	1.399 (3)
N3—H3	0.8600	C12—H12	0.9300
C1—C2	1.390 (4)	C13—C14	1.387 (3)
C1—C6	1.391 (4)	C14—C15	1.390 (3)
C1—H1	0.9300	C14—H14	0.9300
C2—C3	1.376 (4)	C15—H15	0.9300
C2—H2	0.9300	C16—C17	1.516 (3)
C3—C4	1.375 (5)	C17—H17A	0.9600
C4—C5	1.380 (4)	C17—H17B	0.9600
C4—H4	0.9300	C17—H17C	0.9600
			
C8—O1—C7	102.84 (16)	O2—C9—H9A	110.1
C10—O2—C9	115.84 (15)	C8—C9—H9A	110.1
C7—N1—N2	105.90 (18)	O2—C9—H9B	110.1
C8—N2—N1	105.93 (18)	C8—C9—H9B	110.1
C16—N3—C13	128.78 (19)	H9A—C9—H9B	108.4
C16—N3—H3	115.6	C11—C10—O2	115.99 (17)
C13—N3—H3	115.6	C11—C10—C15	119.16 (19)
C2—C1—C6	119.5 (2)	O2—C10—C15	124.84 (18)
C2—C1—H1	120.3	C10—C11—C12	121.27 (18)
C6—C1—H1	120.3	C10—C11—H11	119.4
C3—C2—C1	119.2 (3)	C12—C11—H11	119.4
C3—C2—H2	120.4	C11—C12—C13	119.49 (18)
C1—C2—H2	120.4	C11—C12—H12	120.3
C4—C3—C2	122.1 (3)	C13—C12—H12	120.3
C4—C3—Cl1	118.5 (3)	C14—C13—C12	118.97 (19)
C2—C3—Cl1	119.4 (3)	C14—C13—N3	117.19 (18)
C3—C4—C5	118.8 (3)	C12—C13—N3	123.84 (18)
C3—C4—H4	120.6	C13—C14—C15	120.99 (18)
C5—C4—H4	120.6	C13—C14—H14	119.5
C4—C5—C6	120.4 (3)	C15—C14—H14	119.5
C4—C5—H5	119.8	C10—C15—C14	120.03 (18)
C6—C5—H5	119.8	C10—C15—H15	120.0
C5—C6—C1	120.0 (2)	C14—C15—H15	120.0
C5—C6—C7	119.0 (2)	O3—C16—N3	122.8 (2)
C1—C6—C7	120.9 (2)	O3—C16—C17	121.7 (2)
N1—C7—O1	112.35 (19)	N3—C16—C17	115.5 (2)
N1—C7—C6	128.0 (2)	C16—C17—H17A	109.5
O1—C7—C6	119.63 (19)	C16—C17—H17B	109.5
N2—C8—O1	112.97 (18)	H17A—C17—H17B	109.5
N2—C8—C9	130.30 (19)	C16—C17—H17C	109.5
O1—C8—C9	116.72 (17)	H17A—C17—H17C	109.5
O2—C9—C8	107.97 (17)	H17B—C17—H17C	109.5
			
C7—N1—N2—C8	0.6 (3)	C7—O1—C8—N2	−0.2 (2)
C6—C1—C2—C3	0.5 (5)	C7—O1—C8—C9	178.65 (17)
C1—C2—C3—C4	0.6 (5)	C10—O2—C9—C8	179.63 (16)
C1—C2—C3—Cl1	−179.1 (2)	N2—C8—C9—O2	−8.3 (3)
C2—C3—C4—C5	−0.7 (5)	O1—C8—C9—O2	173.06 (16)
Cl1—C3—C4—C5	178.9 (2)	C9—O2—C10—C11	−179.13 (17)
C3—C4—C5—C6	−0.1 (5)	C9—O2—C10—C15	2.1 (3)
C4—C5—C6—C1	1.1 (4)	O2—C10—C11—C12	178.85 (18)
C4—C5—C6—C7	179.9 (2)	C15—C10—C11—C12	−2.3 (3)
C2—C1—C6—C5	−1.3 (4)	C10—C11—C12—C13	−0.1 (3)
C2—C1—C6—C7	179.9 (2)	C11—C12—C13—C14	2.3 (3)
N2—N1—C7—O1	−0.8 (3)	C11—C12—C13—N3	−178.20 (19)
N2—N1—C7—C6	179.2 (2)	C16—N3—C13—C14	−172.9 (2)
C8—O1—C7—N1	0.6 (2)	C16—N3—C13—C12	7.6 (4)
C8—O1—C7—C6	−179.32 (18)	C12—C13—C14—C15	−2.1 (3)
C5—C6—C7—N1	−3.4 (4)	N3—C13—C14—C15	178.33 (19)
C1—C6—C7—N1	175.5 (2)	C11—C10—C15—C14	2.5 (3)
C5—C6—C7—O1	176.6 (2)	O2—C10—C15—C14	−178.79 (18)
C1—C6—C7—O1	−4.6 (3)	C13—C14—C15—C10	−0.2 (3)
N1—N2—C8—O1	−0.3 (2)	C13—N3—C16—O3	−4.4 (4)
N1—N2—C8—C9	−178.9 (2)	C13—N3—C16—C17	175.4 (2)

### N-(4-{[5-(4-Chlorophenyl)-1,3,4-oxadiazol-2-yl]methoxy}phenyl)acetamide (II) Hydrogen-bond geometry (Å, º)

**Table:**

*D*—H···*A*	*D*—H	H···*A*	*D*···*A*	*D*—H···*A*
C1—H1···O1	0.93	2.54	2.853 (3)	100
N3—H3···N2^i^	0.86	2.55	3.377 (3)	161
C1—H1···O3^i^	0.93	2.46	3.359 (3)	163
C14—H14···N2^i^	0.93	2.59	3.443 (3)	152
C17—H17*C*···O3^ii^	0.96	2.56	3.357 (4)	140

Symmetry codes: (i) −*x*+3/2, *y*+1/2, −*z*+1/2; (ii) *x*, −*y*, *z*+1/2.
